# Suppression of the metastatic spread of breast cancer by DN10764 (AZD7762)-mediated inhibition of AXL signaling

**DOI:** 10.18632/oncotarget.13088

**Published:** 2016-11-04

**Authors:** Joon-Suk Park, ChuHee Lee, Hyun-Kyoung Kim, Dayea Kim, Jung Beom Son, Eunhwa Ko, Joong-Heui Cho, Nam-Doo Kim, Hong-Yan Nan, Choong-Yong Kim, Sukkyoon Yoon, Sun-Hwa Lee, Hwan Geun Choi

**Affiliations:** ^1^ Laboratory Animal Center, Daegu-Gyeongbuk Medical Innovation Foundation, Daegu, South Korea; ^2^ Department of Biochemistry and Molecular Biology, School of Medicine, Yeungnam University, Daegu, South Korea; ^3^ New Drug Development Center, Daegu-Gyeongbuk Medical Innovation Foundation, Daegu, South Korea

**Keywords:** breast cancer, metastasis, AXL, signal transduction, kinase inhibitor

## Abstract

Breast cancer is the most common malignant disease occurring in women and represents a substantial proportion of the global cancer burden. In these patients, metastasis but not the primary tumor is the main cause of breast cancer-related deaths. Here, we report the novel finding that DN10764 (AZD7762, a selective inhibitor of checkpoint kinases 1 and 2) can suppress breast cancer metastasis. In breast cancer cells, DN10764 inhibited cell proliferation and GAS6-mediated AXL signaling, consequently resulting in suppressed migration and invasion. In addition, DN10764 induced caspase 3/7-mediated apoptosis in breast cancer cells and inhibited tube formation of human umbilical vein endothelial cells. Finally, DN10764 significantly suppressed the tumor growth and metastasis of breast cancer cells in *in vivo* metastasis models. Taken together, these data suggest that therapeutic strategies targeting AXL in combination with systemic therapies could improve responses to anti-cancer therapies and reduce breast cancer recurrence and metastases.

## INTRODUCTION

Breast cancer is the most common malignant disease affecting women and contributes substantially to the global cancer burden [6, 22], and metastasis is the main cause of breast cancer-related deaths [25].

AXL belongs to the recently identified TYRO-3, AXL, MERTK (TAM) receptor tyrosine kinase (RTK) family [26]. Members of TAM subfamily of RTKs have the same extracellular domains (2 immunoglobulin-like domains and 2 fibronectin type III domains) and cytoplasmic domain, which has kinase activity [3, 9, 26]. The growth arrest-specific 6 (GAS6) protein serves as a common ligand for each TAM kinase and shows highest affinity for AXL, followed by TYRO-3, and finally MERTK. Upon GAS6 binding, AXL homodimerizes and subsequently induces several downstream signaling pathways involved in cell proliferation, migration, invasion, anti-apoptosis, angiogenesis, metastasis, and therapeutic resistance [3, 9, 18, 26]. Upregulation of AXL and its major ligand GAS6, has been reported in a wide variety of cancer cell lines as well as in cancer specimens from patients with breast cancer, acute leukemia, colorectal cancer, lung cancer, melanoma, ovarian cancer, or prostate cancer, among others [11]. In primary breast cancers, AXL expression independently predicts poor overall patient survival, and AXL expression is enhanced in the metastatic lesions of matched patients [8]. In addition, AXL expression is required for invasiveness, growth, and metastasis in *in vivo* breast cancer models [8, 10]. Thus, AXL has been proposed a very promising target for the development of anti-metastatic breast cancer therapy [8, 10, 28].

Several studies are currently ongoing to develop effective AXL inhibitors, including specific monoclonal antibodies, recombinant extracellular domains that function as “ligand traps,” or small-molecule kinase inhibitors [9, 16]. BGB324 (formerly known as R428) is a first-in-class, highly selective small-molecule AXL inhibitor that is currently in Phase I clinical trials to assess its clinical responses in patients with acute myeloid lymphoma and non-small cell lung cancer (NSCLC) [3, 21].

DN10764 (also known as AZD7762) was previously characterized as a selective inhibitor of checkpoint kinases 1 and 2 (Chk1 and Chk2) [12, 14, 15, 17, 27]. Here, we report a previously unidentified activity of DN10764 against AXL. In breast cancer cells, DN10764 was found to inhibit cell proliferation and GAS6-mediated AXL signaling pathways, resulting in the suppression of migration and invasion. In addition, DN10764 induced caspase 3/7-mediated apoptosis in breast cancer cells and inhibited tube formation of human umbilical vein endothelial cells. Furthermore, DN10764 delayed the metastatic progression of breast cancer cells in *in vivo* metastasis-prevention models.

## RESULTS

### Identification of DN10764 as a potential inhibitor of TAM family RTKs

Previous data highlighted AXL as a target kinase of DN10764 [17]. In addition, data from the publicly available Library of Integrated Network-based Cellular Signature (LINCS) KINOMEscan screen (http://lincs.hms.harvard.edu/db/datasets/20027/) suggested that DN10764 is probably a strong hit against TAM family RTKs at 10 μM. Based on these publicly available data, we independently determined the binding constants (Kds) of DN10764 against human AXL, MERTK, and TYRO-3 using KINOMEscan screening technology (DiscoveRx). As shown in Supplementary Figure S1, DN10764 exhibited relatively strong affinity for AXL (Kd = 26 nM) and MERTK (Kd = 5.5 nM), compared with the affinity of DN10764 for TYRO-3 (Kd = 1050 nM). *In vitro* biochemical enzyme-inhibition assays confirmed that DN10764 profoundly inhibited AXL, MERTK, and TYRO-3 with the IC_50_ values of 4.0 nM, 1.87 nM, and 15.6 nM, respectively (Reaction Biology Corporation; Supplementary Figure S2). Taken together, these data strongly suggested that DN10764 can potentially be developed as a selective inhibitor of members of the TAM family of RTKs, especially against AXL and MERTK.

### DN10764 inhibits the proliferation of human breast adenocarcinoma cells

**Figure 1:**
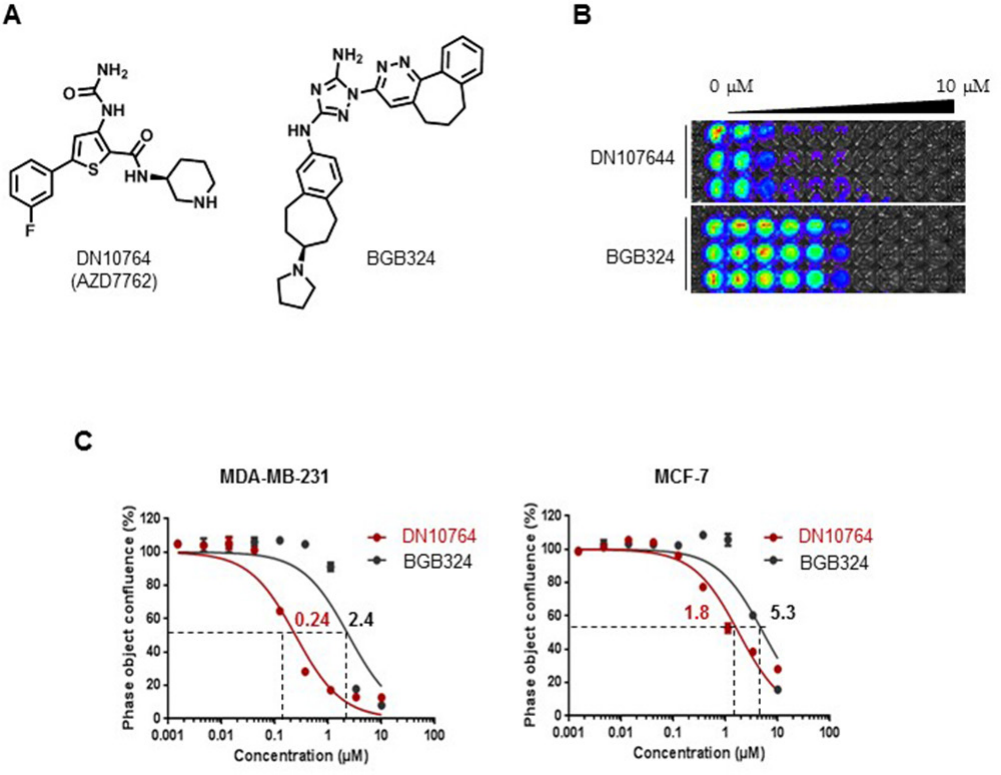
Inhibition of cell proliferation by DN10764 **A.** Chemical structures of DN10764 (AZD7762) and BGB324. MDA-MD-231-luc2-tdTomato cells or MCF7 were seeded in triplicate wells and subsequently treated with medium containing either DN10764 or BGB324 at 10 concentration in a 3-fold serial dilution series (0–10 μM). At 72 h post-treatment, bioluminescent images were taken **B.** or dose-dependent confluency was assessed using the IncuCyte™ ZOOM Imaging System followed by calculating IC_50_ values **C.**

Because cell-free biochemical enzymatic assays do not always correlate with cellular inhibition, the effect of DN10764 on the proliferation of cancer cells was next investigated. The MDA-MB-231 triple-negative breast cancer cell line was chosen for this study because it is well demonstrated that AXL overexpression in this cell line confers aggressive cell behaviors [28]. The MDA-MB-231-luc2-tdTomato cell line, which was derived from MDA-MB-231 cells by stably overexpressing both the luciferase and tdTomato gene, was treated with the indicated concentrations of either DN10764 or BGB324 (Figure 1A) [10, 21]. After 72 h, cell proliferation was monitored for luminescence signals following Luciferin treatment. As shown in Figure 1B, both DN10764 and BGB324 dose-dependently inhibited the proliferation of MDA-MB-231-luc2-tdTomato cells. However, DN10764 more potently inhibited the proliferation of MDA-MB-231-luc2-tdTomato cells than BGB324. We confirmed these results by monitoring cell proliferation in real-time for 72 h using the IncuCyte FLR Imaging System, which revealed IC_50_ values of 0.24 μM for DN10764 and 2.4 μM for BGB324 in MDA-MB-231 cells (Figure 1C left). This anti-proliferative activity of DN10764 was less potent in MCF7 cell line, an AXL-negative breast cancer cell line (Figure 1C right). In addition, we found that Hs578T breast cancer cell line expressing AXL was more sensitive to the anti-proliferative effect of DN10764 than two other AXL-negative breast cancer cell lines such as SK-BR-3 and T47D (Supplementary Figure S3A). Finally, we further confirmed that DN10764 exerts its anti-proliferative effect by targeting AXL using siRNA specific to AXL (siAxl). We found that siAxl substantially decreased AXL expression compared with control siRNA (siCon), which resulted in the augmentation of inhibitory effect of DN10764 on cell proliferation (Supplementary Figure S3B). Taken together, these results clearly demonstrated that DN10764 impedes cell proliferation by targeting AXL.

### DN10764 inhibited the GAS6-induced AXL signaling pathway

**Figure 2:**
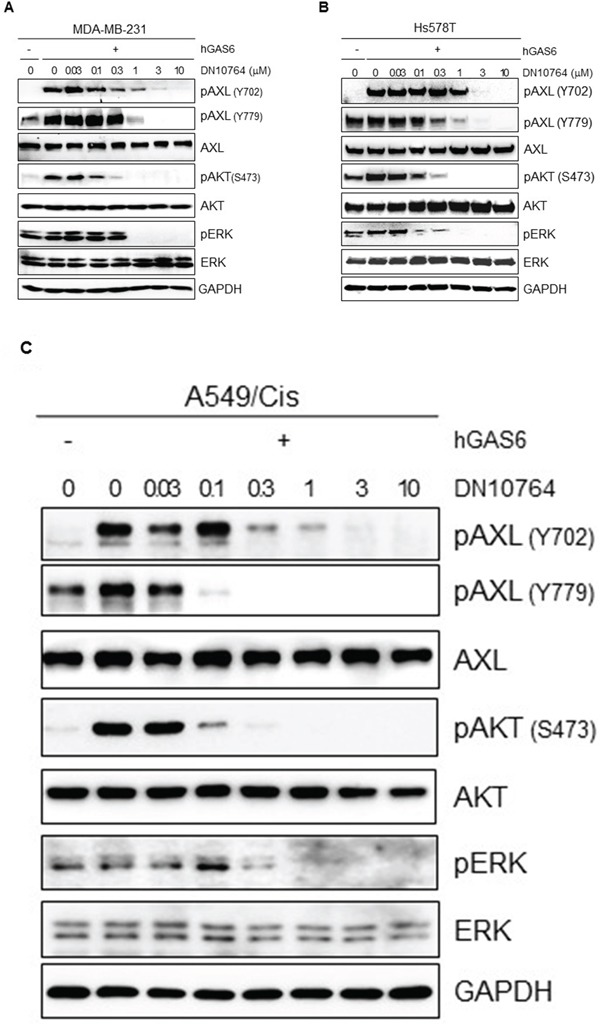
Inhibition of GAS6-mediated AXL signaling pathways MDA-MD-231-luc2-tdTomato cells **A.** or Hs587T cells **B.** or A549/Cis cells **C.** were starved overnight and then pre-treated with for 1 h with DN10764 before GAS6 stimulation. Equal amounts of protein from cell lysates were analyzed by western blotting with the indicated antibodies.

Several different mechanisms can induce activation of AXL signaling pathways, including GAS6-driven ligand-dependent AXL dimerization, ligand-independent homodimerization between 2 AXL monomers, and heterometric dimerization between AXL and a non-TAM receptor [26]. GAS6 binding to the extracellular domain of AXL induces autophosphorylation of tyrosine residues on the intracellular tyrosine kinase domain of AXL, including tyrosines 698, 702, 779, 821, and 866, as well as subsequent activation of MAPK/ERK and PI3K/AKT signaling pathways, which in turn modulate numerous cellular activities including cell survival, proliferation, migration, and invasion [9, 26]. Therefore, we investigated the effect of DN10764 on GAS6-stimulated AXL signaling pathways. To do this, MDA-MB-231 cells were pre-treated with the indicated concentrations of DN10764 for 1 h before stimulation with GAS6. As shown in Figure 2A, we found that MDA-MB-231 cells showed constitutive phosphorylation of AXL at tyrosine residue 779 (Y779), as well as ERK. GAS6 treatment induced AXL phosphorylation at tyrosine residue 702 (Y702) and AKT activation. However, DN10764 pretreatment resulted in a dose-dependent decrease of GAS6-induced AXL phosphorylation at both Y702 and Y779. The overall level of AXL expression was not changed by DN10764 treatment. Moreover, downstream activation of AKT and ERK was consequently inhibited by DN10764. As shown in Figure 2B and 2C, a similar inhibitory effect of DN10764 on GAS6-induced AXL signaling was observed in the Hs578T cell line, another AXL-expressing breast cancer cell line, and in the A549/C is cell line, which is a cisplatin-resistant derivative of the human A549 lung cancer cell line that expresses significantly more AXL compared to the parental A549 cell line [11]. These results clearly demonstrated that DN10764 can inhibit GAS6-induced AXL signaling pathways.

### DN10764 induced apoptosis

**Figure 3:**
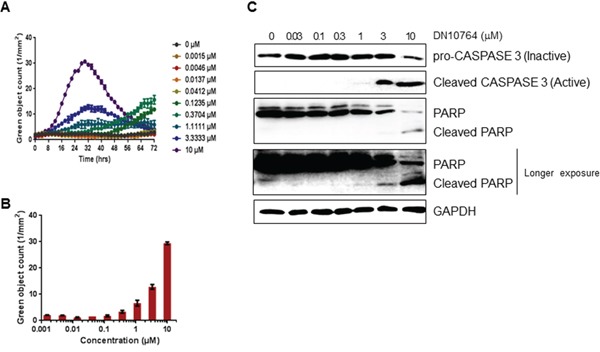
Apoptosis induction by DN10764 **A** and **B.** Activation of caspase 3/7 was quantified using live-cell, time-lapse fluorescent imaging and the IncuCyte™ FLR fluorescent Object Counting Algorithm. All results are shown as the mean standard error of n > 2 replicates. **C.** Equal amounts of protein from cell lysates prepared at 32 h post-treatment were analyzed by western blotting with the indicated antibodies.

To examine whether DN10764 inhibits the proliferation of MDA-MB-231 cells by triggering apoptosis, we monitored the activation of caspase 3/7 activity in real time using the IncuCyte FLR imaging system. As shown in Figure 3A and 3B, caspase 3/7 activity increased in time- and dose-dependent manners over a 32-h treatment period. Therefore, we next examined the level of active pro-caspase 3 protein by western blot analysis. As shown in Figure 3C, the level of inactive pro-caspase 3 was reduced, while the level of active caspase 3 was dose-dependently increased by DN10764 by the end of the 32-h treatment time point. To confirm the function of caspase 3 activity, we examined the total expression level of the poly (ADP-ribose) polymerase (PARP) protein, a substrate of active caspase 3. We found that DN10764 treatment dose-dependently reduced total PARP protein expression, but induced cleaved PARP production. These results clearly showed that DN10764 induced a caspase 3/7-mediated apoptotic pathway in MDA-MB-231 cells by inhibiting anti-apoptotic function of the AXL signaling pathway.

### DN10764 inhibited migration and invasion of human breast adenocarcinoma cells

**Figure 4:**
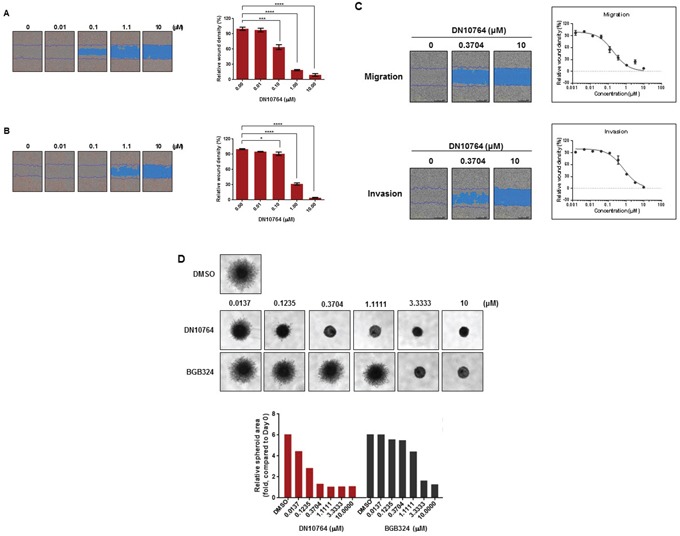
DN10764-mediated suppression of migration and invasion DN10764 significantly inhibited migration and invasion of MDA-MB-231 cells in the absence **A** and **B.** and presence **C.** of mitomycin **C.** The relative wound widths for the migration and invasion assays were calculated at 24 h post-incubation using IncuCyte software. (Right panel) Data are expressed as the mean ± SEM of duplicate samples. One-way ANOVA, with the Turkey–Kramer multiple comparisons test was used. *P < 0.05; ***P < 0.001; ****P < 0.0001. **D.** MDA-MD-231 cells were cultured as 3D spheroids, and images of stellate structure formation were taken at 42 h post-drug treatment. (Lower panel) Relative spheroid areas were measured using Image J software.

Two cellular activities modulated by the AXL signaling pathways are cellular migration and invasion [8, 10, 28]. Thus, we examined whether DN10764 could affect cellular migration and/or invasion. To this end, 2-dimensional cell monolayer wound-healing assays were performed. We found that MDA-MB-231 cells migrated or invaded into the wound at a high rate in the absence of DN10764, closing the wound within 12 h (data not shown). However, the migration or invasion of MDA-MB-231 cells was significantly inhibited in the presence of DN10764 in a dose-dependent manner (Figure 4A and 4B). To distinguish migration from proliferation, MDA-MB-231 cells were treated with mitomycin C for 2 h prior to the wound-healing assay. As demonstrated in Figure 4C, DN10764 inhibited migration and invasion in the presence of mitomycin C, further confirming that the inhibitory effect of DN10764 on cellular migration and invasion was independent of proliferation. Inhibition of invasion by DN10764 was additionally confirmed using a 3-dimensional (3D) spheroid culture system. As shown in Figure 4D, 3D-cultured MDA-MB-231 cells formed large spheroids, with stellate projection structures invading the surrounding matrix in DMSO-treated negative control cells, whereas they grew as relatively small, round spheroids without stellate projections in the presence of either DN10764 or BGB324. Compared to the effect of BGB324, the dose-dependent inhibition of stellate structure formation was greater with DN10764 (Figure 4D). Taken together, these results suggested that DN10764 can inhibit cellular motility and invasivity, potentially by targeting AXL signaling.

### DN10764 treatment suppressed tube formation

**Figure 5:**
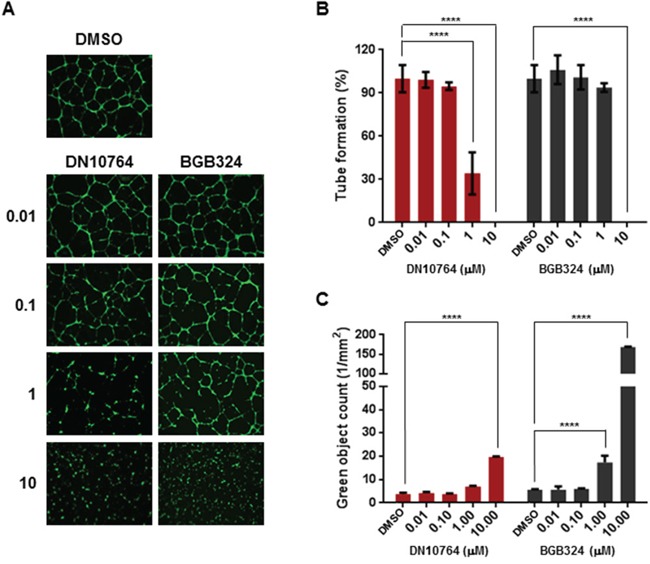
DN10764-mediated inhibition of tube formation **A.** Representative images of HUVECs forming capillary-like structures. HUVECs were treated with the indicated drugs for 16 h in the presence of VEGF. Formation of capillary-like structure was then visualized by staining with Calcein AM. **B.** Tube formation was determined by manually counting the number of branches/fields formed by HUVECS. Data are expressed as the mean ± SEM of 3 randomly selected fields. One-way ANOVA; ****P < 0.0001. **C.** Cytotoxicity was measured using YOYO-1, as described in the Materials and Methods section. Data are expressed as the mean ± SEM of duplicate samples. One-way ANOVA: ****P < 0.0001.

Angiogenesis plays an important role in tumor progression, whereby vascular endothelial cells gain the ability to proliferate off of and extend from existing vessels [3]. It is reported that AXL is expressed in vascular endothelial cells [7, 13, 20]. To assess whether AXL inhibition can affect angiogenesis, we conducted a tube-formation assay *in vitro*, using HUVECs. As shown in Figure 5A and 5B, HUVECs treated with DMSO formed a network of cell clusters connected by long, multicellular processes leading to the formation of tube-like structures, whereas treatment with either DN10764 or BGB324 resulted in marked inhibition of this phenotype. To ensure that the inhibitory effect of drugs on tube formation was not due to cytotoxicity, the cytotoxic effects of the drugs were measured using the cell-impermeable dye YOYO-1. We found that DN10764 was less cytotoxic than BGB324 against HUVECs at 1 μM, indicating that the inhibition of tube formation by DN10764 potentially resulted from the inhibition of AXL signaling rather than cytotoxicity at this concentration (Figure 5C). These results implied that DN10764 can exert its anti-angiogenic activity without a drastic reduction of cell viability.

### DN10764 suppressed metastasis *in vivo*

**Figure 6:**
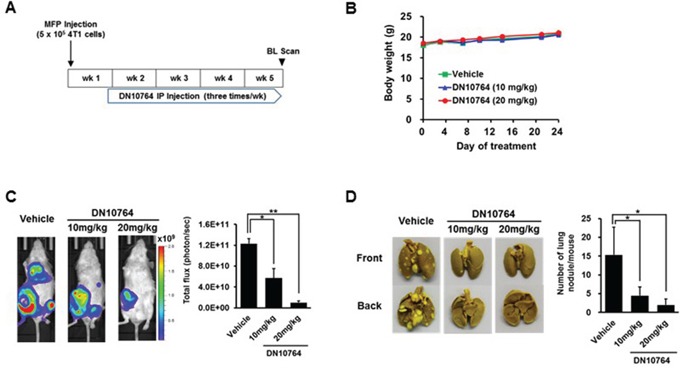
DN10764-meidated suppression of breast cancer metastasis **A.** Scheme of DN10764 treatment. 4T1-luc cells were injected into the fourth mammary fat pad of each mouse under anesthesia. At 6 days after injection, intraperitoneal treatment was initiated with DN10764 (10 mg/kg or 20 mg/kg) or vehicle control, followed by injecting three times per week for 4 weeks. **B.** Body weights of mice treated with DN10764. **C.** Representative whole body bioluminescent images. Serial images were obtained from all animals, and the mean photon flux relative to peak signal was determined. **D.** Tumor nodules observed on the surface of the lungs. Student's *t*-test; *P < 0.05, **P < 0.01.

To evaluate the effect of AXL inhibition on the development of metastasis, two *in vivo* metastasis models were used in this study. As shown in the orthotopic metastasis model using 4T1 cell line (a mouse breast cancer cell line) carrying luciferase gene, DN10764 treatment significantly suppressed the progression of growth (Figure 6C) as well as lung metastasis (Figure 6D) in a dose-dependent manner. These results were further confirmed in another metastasis model, in which mice were pre-pretreated with DN10764 2 h prior to the injection of MDA-MB-231-luc2-tdTomato cells into the arterial circulation of nude mice via intracardiac injection. As shown in Supplementary Figure S4, compared with vehicle-injected mice, the average bioluminescence at day 43 decreased by 24% or 40% for mice treated with 10 or 20 mg/kg DN1076, respectively. Two dosing regimens (10 mg/kg and 20 mg/kg) did not affect the average animal body weight compared with vehicle groups (Figure 6B and Supplementary Figure S4B). Taken together, these results clearly demonstrated that DN10764 suppressed *in vivo* tumor progression and metastasis of breast cancer cells.

## DISCUSSION

DN10764 was previously developed as a novel Chk1 inhibitor by AstraZeneca [27]. It has been shown that AZD7762 not only sensitizes various tumor cells to DNA damage *in vitro*, but also retards tumor progression *in vivo* in response to radiation or DNA-damaging agents [2, 4, 12, 14, 23, 27]. In this study, we examined the potential of DN10764 as an AXL inhibitor, based on publicly available data from the LINCS KINOMEscan screen project, and found that DN10764 interfered with AXL-mediated cellular events such as cell proliferation, anti-apoptosis, cellular migration and invasion, and *in vivo* breast cancer metastasis. These results suggested the clinical potential of DN10764 for controlling the metastatic progression of breast cancer occurring through AXL overexpression.

We found that DN10764 exhibited more potent anti-proliferative activity than did BGB324, a first-in-class, highly selective small-molecule AXL inhibitor. This may be due to intrinsic differences between these compounds, such as cell permeability and the stability within cells. Moreover, we cannot exclude the possibility that DN10764 may target more kinases than BGB324 in cells. In fact, data from published studies suggest that DN10764 might show activity against many other kinases [17]. Therefore, it is possible that inhibition of additional kinase-mediated signaling may have occurred concomitantly with the AXL inhibition by DN10764. However, examination of GAS6-induced AXL signaling events clearly showed that DN10764 dose-dependently inhibits GAS6-induced activation of AXL and subsequent AKT and ERK signaling.

Recent findings have demonstrated that AXL potently promotes invasion and metastasis in experimental models, and AXL expression correlates with poor outcomes with breast cancer patients [8, 10, 28]. In this study, we showed that DN10764 significantly reduced the migration and invasion of breast cancer cells *in vitro* and breast cancer metastasis *in vivo*. Previous investigators proposed that vimentin, an intermediate filament protein, functionally contributes to EMT activation and is required for AXL upregulation, which contributes to the lung extravasation of breast cancer cells in mice [24]. Moreover, data from a recent study demonstrated that AXL phosphorylates and interacts with Elmo in breast cancer cells, leading to induction of the RAC pathway and, consequently, the promotion of cell invasion. Similar to AXL inhibition, it has shown that Elmo knockdown suppressed the proliferation and invasion of breast cancer cells [1]. Thus, it remains to be determined whether EMT and RAC pathways are also inhibited by DN10764 treatment, which should be addressed in future studies.

Previous studies have reported functional roles for AXL in vascular endothelial cells [7, 13, 20]. Results from our tube-formation assay using HUVECs revealed that, compared to BGB324, DN10764 significantly suppressed tube formation at a concentration of 1 μM. Concurrent cytotoxicity assays performed in this study further implied that unlike BGB324, drastic suppression of tube formation by DN10764 at this concentration occurs without cell toxicity.

Data from our two independent *in vivo* metastasis models showed that, compared to the vehicle-treated group, overall tumor volumes were reduced in the DN10764-treated groups, although metastasis still occurred in the DN10764-treated groups. These results indicate that DN10764 might exert its anti-metastatic activity on early metastatic steps, such as cancer cell adherence to the vascular endothelium and extravasation. Interestingly, however, we found that lower dosing of DN10764 (rather than higher dosing) resulted in better suppression of metastatic spread in the intracardiac metastasis model. As the TAM RTKs are known to negatively regulate inflammatory immune responses [5, 9], high DN10764 dosing may have targeted both AXL and MERTK, resulting in hyperinflammation *in vivo*, ultimately contributing to the promotion of tumor progression and metastasis. Recent data demonstrated that a small-molecule inhibitor of the TAM RTKs consistently reduced melanoma and mammary tumor metastasis in the lung and liver in an NK cell-dependent manner, without any serious side effects [19]. Thus, determining whether TAM RTK inhibitors are useful for anti-cancer therapy will require a thorough analysis of the risks and benefits of removing a homeostatic check on innate immune inflammatory responses, as well as the optimal dose and duration of therapy.

In summary, herein we report that, unlike its previously published activity as a sensitizer to DNA damage, DN10764 as a single agent can efficiently inhibit cancer cell proliferation. This anti-proliferative activity is through the suppression of cellular AXL signaling events including anti-apoptosis, migration, invasion, and angiogenesis. Furthermore, given the fact that AXL is involved in acquired drug resistance of many solid tumors including NSCLC, leukemia, breast cancer, ovarian cancer, and cancers of the gastrointestinal tract [18], development of a selective AXL inhibitor will help the successful management of advanced cancer patients with acquired resistance to targeted therapies.

## MATERIALS AND METHODS

### *In vitro* compound screening

Binding constants (Kds) of DN10764 against the human AXL, MERTK, and TYRO-3 kinases were determined in duplicate mode by DiscoveRx. The enzyme activities (IC_50_ values) of these kinases were determined by Reaction Biology Corporation, using 10 concentrations in a 3-fold dilution series of DN10764 starting at 10 μM.

### Reagents

Both DN10764 and BGB324 were purchased from Selleck Chemicals. The following antibodies were used in this study. Anti-AXL (#AF154) and anti-phospho-AXL (Y779, #AF2228) antibodies were purchased from R&D Systems. Anti-phospho-AKT (S473, #4060), anti-AKT (#4685), anti-phospho-ERK (#9106), anti-ERK (#4695), anti-PARP (#9542), anti-cleaved PARP (#5625), anti-pro-CASPASE 3 (#9665), anti-CASPASE 3 (#9661), and anti-phospho-AXL (Y702, #5724) antibodies were purchased from Cell Signaling Technology. An anti-GAPDH antibody (sc-25778) was purchased from Santa Cruz Biotechnology, Inc. Human GAS6 was purchased from R&D Systems.

### Cell culture

The MDA-MB-231-luc2-tdTomato cell line was purchased from Perkin Elmer Inc. and grown in EMEM (American Type Culture Collection [ATCC]) containing 10% FBS, without antibiotics. HUVECs were purchased from the ATCC and grown in complete Vascular Cell Basal Medium (ATCC #PCS-100-030) supplemented with FBS (2%), bovine brain extract (0.2%), hydrocortisone hemisuccinate (1 μg/ml), endothelial growth factor (10 ng/ml), heparin sulfate (0.75 U/ml), L-glutamine (10 mM), and ascorbic acid (50 μg/ml). Hs578T, SK-BR-3, and T47D cell lines were purchased from Korean Cell line Bank (Seoul Korea) and grown in RPMI-1640 (Gibco BRL) containing 10% FBS, 2 mM L-glutamine, 10 U/ml penicillin and 10 g/ml streptomycin.

### Western blot analysis

MDA-MD-231-luc2-tdTomato cells were starved in medium containing 0.5% FBS overnight and then pretreated with the indicated concentration of DN10764 for 1 h before stimulation with human GAS6 (2 μg/ml). Total cell lysates were prepared using RIPA buffer (Thermo Scientific) containing protease inhibitor cocktail. Proteins from the cell lysates (20–40 μg) were separated by SDS-PAGE, and electrotransferred onto polyvinylidene fluoride membranes (Bio-Rad). Western blot analyses were performed as previously described [11].

### Cell proliferation assay

MDA-MB-231-luc2-tdTomato cells (2 × 10^3^/well) were seeded in 96-well plates and allowed to adhere overnight. The cultures were then re-fed with medium containing either DN10764 or BGB324 at 10 concentrations over a 3-fold serial dilution series (0–10 μM). Plates were placed within a micro plate tray in a IncuCyte™ ZOOM Imaging System (Essen Biosciences), equipped with a 10× objective in a CO_2_ incubator at 37°C. Images were collected every 4 h in phase-contrast mode for a period of 72 h. Proliferation was measured using the real-time, phase-contrast images according to the manufacturer's instructions. Cell proliferation was also measured by CCK-8 assay (Dojindo) according to the manufacturer's instructions.

### siRNA transfection

RNA interference silencing was performed to reduce Axl expression. Cells (3 × 10^5^) were seeded in 60-mm culture dishes, grown overnight and then transfected with 50 nM siRNA targeting Axl (sense, 5′-AAGAUUUGGAGAdACACACUGA-3′ and antisense, 5′-UCAGUGUGUUCUCCAAAUCUU-3′) or control siRNA. At 24 h post-transfection, cells were divided and treated with the indicated concentrations of inhibitor or vehicle, respectively. Then, cells were harvested 24 h later to evaluate protein expression and cell proliferation.

### Measurement of caspase activities

Cells were grown overnight to 25–35% confluence in 96-well plates. The medium was then replaced with medium containing the indicated concentrations of DN10764 and 5 μM of the caspase-3/7 apoptosis reagent (Essen Biosciences), which is a cell-permeable, non-fluorescent substrate that can be cleaved by activated caspase 3/7 in cells to release a fluorescent green DNA-labeling dye. Plates were placed within a micro plate tray in a IncuCyte™ ZOOM Imaging System, equipped with a 10× objective in a CO_2_ incubator at 37°C. Real-time phase-contrast and fluorescence images were collected every 4 h for a period of 72 h. Activation of caspase-3/7 was quantified according to the manufacturer's instructions.

### Cytotoxicity assay

HUVECs were grown overnight. The medium was then replaced with fresh medium containing the indicated concentrations of DN10764 and 50 nM of the YOYO-1 Cytotoxicity Reagent (Essen Biosciences), a fluorescent, cell-impermeant cyanine dimer, nucleic acid-staining dye. When added to the culture medium, YOYO-1 fluorescently stains the nuclear DNA of cells that have lost plasma membrane integrity. YOYO-1 fluorescence was then monitored over 16 h and quantified according to the manufacturer's instructions.

### *In vitro* migration/invasion assays

Cells (4 × 10^4^) were seeded and grown overnight to 90–100% confluency in collagen I-coated 96-well ImageLock tissue culture plates (Essen BioScience). Monolayers were wounded according to the manufacturer's instructions. For the migration assay, cells were washed twice with PBS to remove the detached cells and then fed fresh, complete medium containing the indicated concentrations of DN10764. For the invasion assay, cells were covered with 100 μL of medium containing Matrigel (2.5 mg/ml; Promega). Once the Matrigel hardened, the cells were fed with complete medium containing the indicated concentrations of DN10764. During the next 24 h, images of cell migration and invasion were automatically acquired with an IncuCyte™ ZOOM Imaging System at 4-h intervals in phase-contrast mode. To investigate the effect of mitomycin C, cells were pretreated with mitomycin C (10 μg/ml) 2 h prior to the wound-healing assay. The data were analyzed with respect to wound confluence and wound healing was calculated according to the manufacturer's instructions.

### Three-dimensional (3D) cell invasion assay

To confirm the inhibition of invasivity of MDA-MB-231 cells following DN10764 treatment, the Cultrex^®^ BME Cell Invasion Assay (Trevigen) was performed according to the manufacturer's instructions. Briefly, 50 μl of 1× Spheroid Formation ECM containing 3,000 single cells was dispensed into each well of a 96-well plate, followed by incubation in a CO_2_ incubator at 37°C for 72 h. Invasion matrix (50 μl) was then added to each well of the plate. After incubating the plate in a CO_2_ incubator at 37°C for 1 h, the cells were fed with 100 μl of warm culture medium containing the indicated concentrations of DN10764 and then maintained in a CO_2_ incubator at 37°C for an additional 72 h. Spheroid formation in each well was then photographed under a Leica microscope under a 4× objective.

### Vascular tube formation

Twenty-four well plates were coated with growth factor-reduced Matrigel for 30 min at room temperature. HUVECs (4 × 10^4^ cells/well) were mixed with 300 μl of media containing the indicated concentrations of drugs and VEGF (10 ng/ml) and then seeded into each well of a Matrigel-coated 24-well plate. After incubation in a CO_2_ incubator at 37°C for 15 h, the cells were stained with Calcein AM (2 μg/ml) for 30 min at 37°C. Images were taken using an EVOS microscope (Life Technologies) under a 4× objective.

### *In vivo* metastasis model

The study was approved by the Institutional Animal Care and Use of Committee of Daegu-Gyeongbuk Medical Innovation Foundation (DGMIF) and performed in accordance with protocols approved by the Institutional Animal Care and Use of Committee. Six-week-old female athymic nude (BALB/c *nu*/nu) mice were purchased from Orient Bio (Seongnam, Korea) and housed in a specific pathogen-free facility at the Laboratory Animal Center of DGMIF before use. For orthotopic metastasis model, 4T1-luc cells were suspended in an ice-cold 50:50 solution of growth factor reduced Matrigel (BD Biosciences) and HBSS, and then fifty microliters of final cell suspension (5 × 10^5^cells) were introduced into the right fourth mammary fat pad of each mouse (n = 3) under anesthesia using a 30-gauge insulin needle. Intraperitoneal treatment with DN10764 (10 mg/kg or 20 mg/kg) or vehicle control was initiated at 6 days post-tumor cell inoculation, followed by injecting 3 times per week for 4 weeks. At the end of the experiment, the lungs were excised and fixed in Bouin's solution (Sigma). The visible metastatic nodules in the lungs were quantified.

For *in vivo* metastasis-prevention model, MDA-MB-231-ludc3-tdTomato cells (2 × 10^5^) were injected into the left ventricle of the heart of each mouse (n = 5) under anesthesia, which were intraperitoneally pre-treated with DN10764 (10 mg/kg or 20 mg/kg) or vehicle 2 h prior to tumor cell injection. Intraperitoneal injection with DN10764 (10 mg/kg or 20 mg/kg) or vehicle was initiated on day 1 post-inoculation and continued 3 times per week for 6 weeks.

### Bioluminescence imaging

Mice were intraperitoneally administered firefly D-Luciferin potassium salt (Perkin Elmer) at a dose of 150 mg/kg body weight in Dulbecco's phosphate-buffered saline. Bioluminescence images were acquired with the IVIS Lumina system (Perkin Elmer). During image acquisition, anesthesia was maintained with 2% isoflurane. Analysis was performed with Living Image^®^ software by measuring the photon flux (measured in photons/[sec · cm^2^ · steradian using a region of interest manually drawn over the body of the mouse. Signals were measured for approximately 1 h, until they decayed considerably. Serial images were obtained from all animals, and the mean photon flux relative to the peak signal was determined. The metastasis burden was monitored via tri-weekly bioluminescence imaging.

### Statistical analysis

Half-maximal inhibitory concentration (IC_50_) values were determined from dose-response curves by 4-parameter curve fitting. Data are expressed as the mean ± standard error of the mean (SEM) of duplicate samples. To determine statistical significance, 1-way analysis of variance (ANOVA) with the Turkey–Kramer multiple comparisons test was used. *P < 0.05; **P < 0.01; ***P < 0.001; ****; *P* < 0.0001. Comparisons that did not reach statistical significance were not noted.

## SUPPLEMENTARY FIGURES


