# Supplementary material for: Suppression of the metastatic spread of breast cancer by DN10764 (AZD7762)-mediated inhibition of AXL signaling
Source: Oncotarget. 2016 Nov 4;7(50):83308–18. doi: 10.18632/oncotarget.13088 (PMC5347771; doi:10.18632/oncotarget.13088)
Supplement: Supplementary file 1 [file oncotarget-07-83308-s001.pdf]

Suppression of the metastatic spread of breast cancer by DN10764 (AZD7762)-mediated inhibition of AXL signaling

SUPPLEMENTARY FIGURES

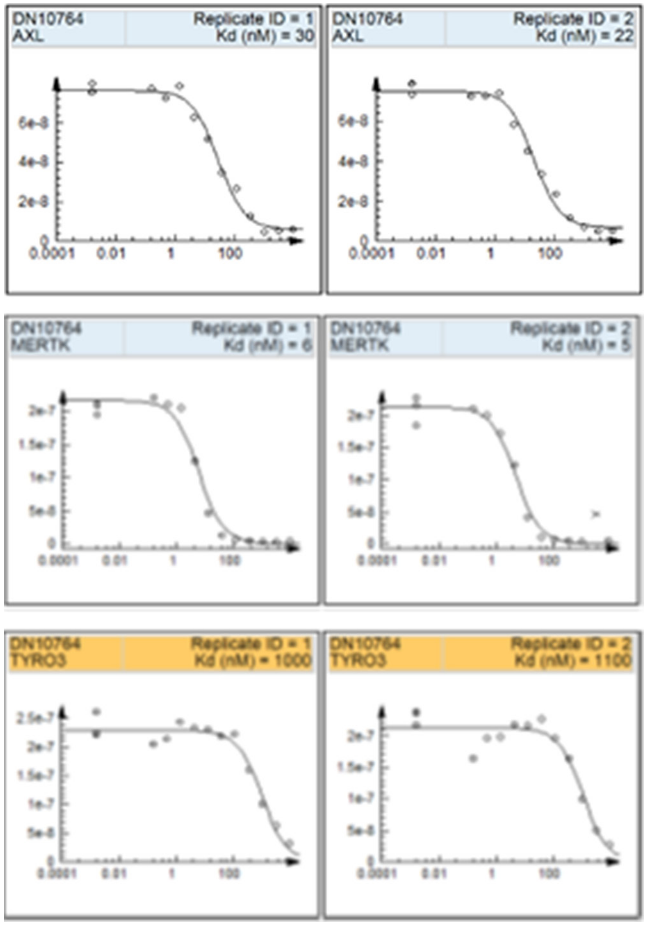

Supplementary Figure S1: Determination of binding constants (Kds). Kd values of DN10764 (AZD7762) were determined against AXL, MERTK, and TYRO3 in duplicate mode by DiscoveRx.

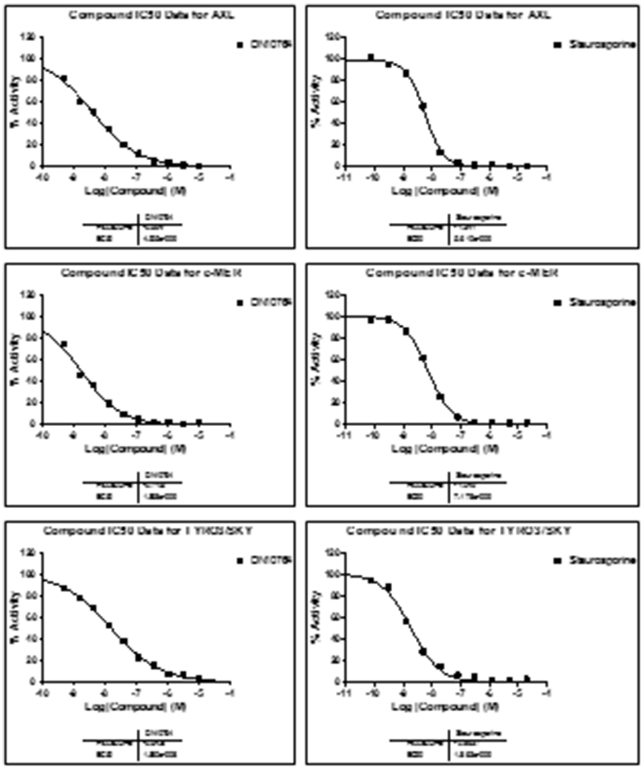

Supplementary Figure S2: *In vitro* enzyme inhibition assay. A cell-free biochemical enzyme inhibition assay was performed against AXL, MERTK, and TYRO3 with ten 3-fold serial dilutions of DN10764 starting at 10 µM by Reaction Biology Corporation.

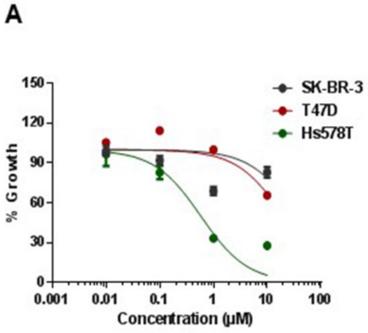

Cell type	IC_{50} (μM)
Hs578T	0.58
SK-BR-3	>10
T47D	>10

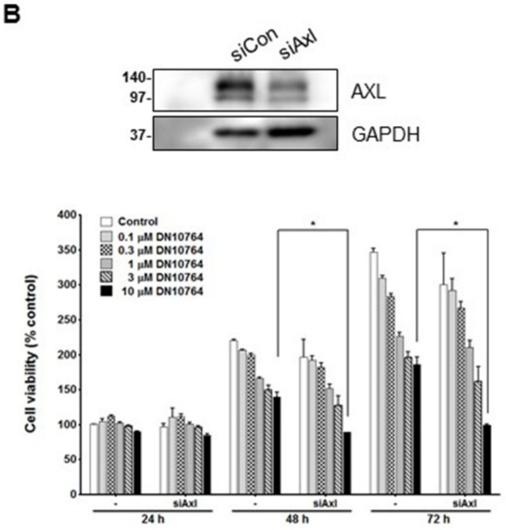

Supplementary Figure S3: DN10764 exerts its anti-proliferative activity of breast cancer cells by targeting AXL. A. The anti-proliferative effect of DN10764 was examined in AXL-positive Hs587T as well as AXL-negative SK-BR-3 and T47D cells. Cells (1×10^3 cells) were seeded in 96 well plates in triplicates and treated with the indicated concentrations of DN10764 for 72 h. **B.** Knock-down of AXL protein level augments the anti-proliferative effect of DN10764. Cells were transfected with AXL specific siRNA (siAxl) or control siRNA (siCon), respectively, and harvested 24 h post transfection, divided into two groups, and grown for the next 24 h in the presence or absence of DN10764. The total cell lysates were prepared and AXL protein levels were determined by Western blot analysis. GAPDH was used as a loading control (top). Cell proliferation was determined using CCK-8 assay kit (bottom). Data are represented as mean ± SD of at least three independent experiments. The asterisks indicate the significant difference compared to the control value (*P<0.05 vs untreated group).

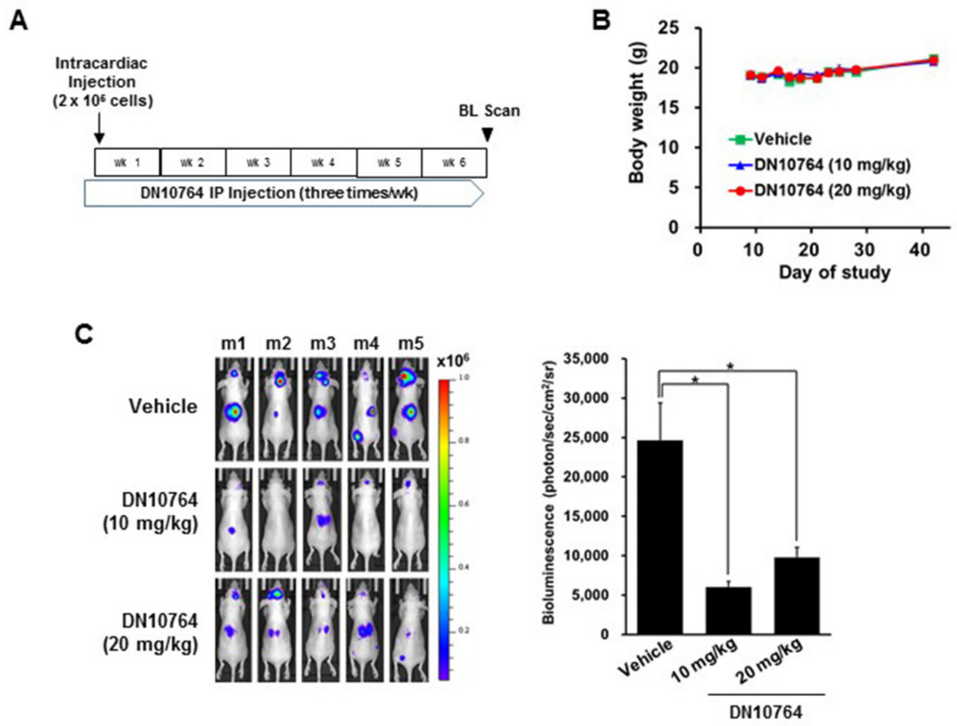

Supplementary Figure S4: Prevention of breast cancer metastasis by DN10764 treatment. A. Scheme of DN10764 treatment. Six-week-old female athymic nude (BALB/c *nu*/nu) mice were intraperitoneally pre-treated with DN10764 (10 mg/kg or 20 mg/kg) or vehicle control 2 h prior to tumor cell injection. MDA-MB-231-luc2-tdTomato cells (2×10^5) were then injected into the left ventricle of the heart of each mouse (n = 5) under anesthesia. Treatment with DN10764 or vehicle control was initiated on day 1 post-tumor cell inoculation, followed by injecting 3 times per week for 6 weeks. **B.** Body weights of mice treated with DN10764. **C.** Bioluminescent images were acquired with an IVIS Lumina system at 43 days post-treatment. (Right panel) Whole-body bioluminescent signals were determined for approximately 1 h, until the signals decayed considerably. Serial images were obtained from all animals, and the mean photon flux relative to peak signal was determined. Student's *t*-test; $^*P < 0.05$
